# Small Cell Lung Cancer Therapeutic Responses Through Fractal Measurements: From Radiology to Mitochondrial Biology

**DOI:** 10.3390/jcm8071038

**Published:** 2019-07-16

**Authors:** Isa Mambetsariev, Tamara Mirzapoiazova, Frances Lennon, Mohit Kumar Jolly, Haiqing Li, Mohd W. Nasser, Lalit Vora, Prakash Kulkarni, Surinder K. Batra, Ravi Salgia

**Affiliations:** 1 City of Hope, Dept. of Medical Oncology and Therapeutics Research, Duarte, CA 91010, USA; 2 Abbott Molecular, Des Plaines, IL 60018, USA; 3 Center for BioSystems Science and Engineering, Indian Institute of Science, Bangalore 560012, India; 4 City of Hope, Center for Informatics, Duarte, CA 91010, USA; 5 City of Hope, Dept. of Computational & Quantitative Medicine, Duarte, CA 91010, USA; 6 University of Nebraska Medical Center, Dept. of Biochemistry and Molecular Biology, Omaha, NE 68198, USA; 7 City of Hope, Dept. of Diagnostic Radiology, Duarte, CA 91010, USA

**Keywords:** small cell lung cancer, radiology, fractal dimension, lacunarity, mitochondria

## Abstract

Small cell lung cancer (SCLC) is an aggressive neuroendocrine disease with an overall 5 year survival rate of ~7%. Although patients tend to respond initially to therapy, therapy-resistant disease inevitably emerges. Unfortunately, there are no validated biomarkers for early-stage SCLC to aid in early detection. Here, we used readouts of lesion image characteristics and cancer morphology that were based on fractal geometry, namely fractal dimension (FD) and lacunarity (LC), as novel biomarkers for SCLC. Scanned tumors of patients before treatment had a high FD and a low LC compared to post treatment, and this effect was reversed after treatment, suggesting that these measurements reflect the initial conditions of the tumor, its growth rate, and the condition of the lung. Fractal analysis of mitochondrial morphology showed that cisplatin-treated cells showed a discernibly decreased LC and an increased FD, as compared with control. However, treatment with mdivi-1, the small molecule that attenuates mitochondrial division, was associated with an increase in FD as compared with control. These data correlated well with the altered metabolic functions of the mitochondria in the diseased state, suggesting that morphological changes in the mitochondria predicate the tumor’s future ability for mitogenesis and motogenesis, which was also observed on the CT scan images. Taken together, FD and LC present ideal tools to differentiate normal tissue from malignant SCLC tissue as a potential diagnostic biomarker for SCLC.

## 1. Introduction

Small cell lung cancer (SCLC) is an aggressive neuroendocrine disease that exhibits rapid growth and early loco-regional and distant metastases [1]. SCLC arises mostly in heavy smokers and accounts for around 13%–15% of all lung cancers [1]. The majority of SCLC patients are diagnosed at late stages, long after the patient begins to exhibit symptoms. For the past 25 years, the standard of care for SCLC has been chemotherapy in combination with radiation for limited stage disease patients, and chemotherapy alone for extensive disease patients. The overall 5 year survival rate in SCLC has also remained constant at 7% [2]. However, more recently, a phase III IMpower133 randomized double-blind study evaluated the addition of a checkpoint inhibitor of programmed death signaling (atezolizumab) to chemotherapy and showed improved benefits with SCLC; while promising, the trial only showed a median overall survival improvement of 2 months [3]. Therefore, early diagnosis is crucial to improving the rate of survival. 

The National Lung Screening Trial demonstrated that screening high-risk patients with low-dose computer tomography (CT) scans led to higher numbers of early-stage adenocarcinomas compared with chest X-ray, and led to a reduction in cancer-specific mortality. However, there was no evidence of mortality improvement or an increase in early-stage diagnoses in SCLC cases [4]. No early screening or early detection methods have been proven to be effective for SCLC [4,5,6]. Consequently, the development of more effective biomarkers for and approaches to SCLC early detection remains a critical and immediate goal in treatment of SCLC. 

Early detection of SCLC can have a profound effect on outcomes for this devastating disease. To date, there are no validated biomarkers for early stage SCLC, and the only known preventative measure for SCLC is smoking abstinence or cessation. Based on our previous work and the work of others [7], we hypothesized that SCLC progression produces highly self-similar growth patterns that generate fractal geometries not easily distinguished by standard radiology measurements. We predicted that these geometries can distinguish benign tissue from SCLC lesions, and that these geometries correlate with tumor aggressiveness. Accordingly, we propose a novel set of measures of lesion image characteristics and cancer morphology that are based on fractal geometry. Our biomarker measurements are based on the concepts of fractal dimension (FD) and lacunarity (LC): the two key fractal geometric parameters that define self-similarity [7,8].

FD is a measure of shape complexity. It ranges from 1 to infinity, with a value of 1 indicating simple geometry such as a line, with higher values corresponding to more complex patterns. The FD of an SCLC lesion quantifies the complexity of its growth pattern and biological aggressiveness [7,8]. On the other hand, LC is a measure of how objects fill space [7,8,9]. It inversely correlates with density and can be interpreted as a measure of the rotational invariance of an object [9]. For instance, in three dimensions, a sphere would have very low LC, whereas a snowflake would have much higher LC. In this study, LC was utilized as a surrogate for spatial heterogeneity of an SCLC lesion. Fractal properties (FD, LC) were employed to characterize the shape and complex properties of SCLC. Non-trivial measures of FD and LC of cancer in mitochondria, tissue histology, and computed tomography (CT) can be correlated with disease progression [7,8]. SCLC evolves as a fractal on multiple scales, from sub-cellular (nm), to cellular (μm), to lesion (mm), and, therefore, measurements of the geometrical properties on these scales could be used as biomarkers for early detection of SCLC and to inform on the aggressiveness of the tumor. This study aims to provide a biological understanding to standard “radiomic” analysis [10] through an approach that is driven by mathematical measures linked to specific biological hypotheses that can be tested in controlled settings. 

## 2. Experimental Section

### 2.1. Cell Culture and Reagents 

Beas-2B, normal transformed human bronchus epithelial cells (ATCC), suspension H69, H82, H526 (ATCC), and adherent SBC3 and SBC5 SCLC cell lines were maintained in RPMI-1640 medium (Corning) supplemented with 10% (v/v) fetal bovine serum (FBS), 1%(v/v) penicillin/streptomycin, and l-glutamine at 37 °C with 5% CO_2_, while DMS114 and DMS273 were maintained in eagle minimum essential medium (E-MEM) (Millipore Sigma, St. Louis, MO, USA) with 10% FBS. The inhibitors for the study cisplatin and mdivi-1 were obtained from Sigma. Mitochondria in live cells were stained with MitoTracker Deep red FM (Life technologies, Carlsbad, CA, USA). Beas-2B cells were grown in tissue-culture-treated plastic (Corning Inc., Corning, NY, USA) for 11 passages. Cells were tested for mycoplasma every 6 months in our laboratory using the MycoProbe Mycoplasma Detection Kit (R&D Systems, #CUL001B, Minneapolis, MN, USA). Short tandem repeat (STR) testing was done before starting the project.

### 2.2. Immunoblotting 

Whole cell lysates were prepared using radioimmunoprecipitation assay (RIPA) lysis buffer. Protein concentration for each sample was measured with Bradford protein assay (Bio-Rad Laboratories #5000201, Hercules, CA, USA). Protein expression was detected by immunoblotting as previously described, using MFN2 (Abcam, ab56889, Cambridge, United Kingdom), DRP1 (Santa Cruz Biotechnology, sc-271583, Dallas, TX, USA), TRAP1 (C8) (Santa Cruz, sc-13134), PGC-1α (Cell Signaling Technology, 2178, Danvers, MA, USA), FIS1 (C10) (Santa Cruz, sc-376469), and pan-actin antibodies (CST, 4968) [11].

### 2.3. Immunofluorescence

Cells were cultured for 24 h on glass coverslips and fixed in 4% paraformaldehyde (PFA) for 10 min at 37 °C. Cells were permeabilized in 0.2% Triton X-100 and blocked in 5% bovine serum albumin (BSA)/ phosphate-buffered saline (PBS). Coverslips were incubated with primary antibody TOM20 (F10) (Santa Cruz, sc-17764) for 60 minutes at room temperature (RT), followed by Alexa-647 conjugated secondary anti-Mouse. Slides were then mounted using Prolog Gold anti-Fade/DAPI mounting media (Life Technologies). Images were acquired using optimal Airyscan parameters, and Airyscan images were post-processed using the Zeiss Zen Black Airyscan processing function (Carl Zeiss Microscopy GmbH, Jena, Germany). 

### 2.4. Tissue Microarray 

The Institutional Review Board at City of Hope (Duarte, CA, USA) approved all of the human subjects research performed under this study, and all subjects were considered exempt consent. All methods were carried out in accordance with the respective approved protocol. In brief, tissue cores (1 mm punch) from biopsied tissue samples were precisely organized into a grid and embedded in paraffin. The paraffin block was cut and the tissue microarray (TMA) was processed for hematoxylin and eosin (H&E) staining. Pathologic diagnosis in these cases was reviewed by at least two experienced pathologists. The H&E stained TMA was scanned using a 3D-Histech Pannoramic SCAN whole slide scanner (3D-Histech, Budapest, Hungary).

### 2.5. Immunohistochemistry

Tissues were stained for H&E and immunohistochemistry (IHC) with primary antibodies (MET (D-4) (Santa Cruz, sc-514148), FAK (CST, 3285), pMET (Y1003) (Abcam, ab193270), and pFAK (Y397) (CST, 8586). Tissue regions were selected at random and analyzed for FD and LC from H&E and IHC images to determine the relationship between the FD and LC values for normal and SCLC tissue.

### 2.6. Fractal Dimension and Lacunarity Analysis 

Tumor tissue images were magnified at 15× magnification from whole slide scanned images for FD and LC analysis. ImageJ plugin FracLac was utilized to convert the images of the tumor tissue to grayscale and to calculate the FD and LC of each sample [12]. The box-counting method in this plugin analyzes the grayscale pattern and returns an intensity fractal dimension based on the differences in pixel density in each box. There were 12 initial grid positions that were used, with box sizes that ranged from 2 × 2 pixels and increased until they reached the maximum size of 45% of the image area. FD was estimated from the regression line of a log–log plot of intensity versus box size for each grid position. FD was then calculated as average of these estimates. LC was calculated based on the number of positive pixels in each box at different box sizes.

### 2.7. Cell Metabolism 

A Seahorse Bioscience XF24 Extracellular Flux Analyzer (Seahorse Bioscience, North Billerica, USA) was used to measure OCR (oxygen consumption rates) and ECAR (extracellular acidification rates). Control and mesothelioma cell lines were maintained in normal complete growth media and seeded onto a gelatin-coated 24 well XF Flux analyzer assay plate at 80,000 cells/well 24 h prior to assay. Cells were switched to serum free XF assay media (Seahorse Biosciences, North Billerica, MA, USA) with 25 mM glucose, 1 mM sodium pyruvate, and 2 mM glutamine (Corning) and placed in a CO_2_-free incubator at least 1 h prior to start of assay. A Seahorse XF24 Analyzer was then used to measure the cellular bioenergetic profile. Each cycle included 3 min of mixing, a 2 min wait, and finally, measurement over 2 min. Four measurements were obtained at baseline and following injection of oligomycin (1 μM; Sigma), FCCP (1 μM; Sigma), and rotenone (1 μM; Sigma). Measurements were normalized to total protein content per well using the Bradford protein assay (Bio-Rad #5000201).

### 2.8. Cytotoxicity Assays

To determine specific cytotoxicity, we used cell-permeable Calcein-AM (Santa Cruz, #CAS14504-34-1). The hydrolysis of Calcein-AM by intracellular esterases produces Calcein, a hydrophilic, strongly fluorescent compound (488 nm/520 nm) that is well-retained in the cell cytoplasm. Cells were seeded in black-walled, 96 well plates and allowed to adhere in normal growth media for 24 h. Cells were then washed once in PBS and the test compounds were added in 1% serum media at the indicated concentrations for 72 h. The medium was then removed and replaced with 100 ul of phenol red-free Opti-MEM containing 2 μM Calcein-AM and incubated for a further 30 min. Samples were measured using a Bio-Tek Synergy multi-detection microplate reader equipped with 485 nm excitation and 535 nm band-pass emission filter (BioTek, Winooski, VT, USA).

### 2.9. Statistical Analysis

The Graph Pad Prism 5.0 software (GraphPad, San Diego, CA, USA) package was used for the statistical analysis. One-way ANOVA with Tukey multiple comparison post-test was used as appropriate for multiple group comparisons. Student’s t-test was used for pairwise comparisons. All tests were two-sided and *p* < 0.05 was considered statistically significant.

## 3. Results

### 3.1. Radiological Quantification of SCLC CT Scans

**Figure 1:**
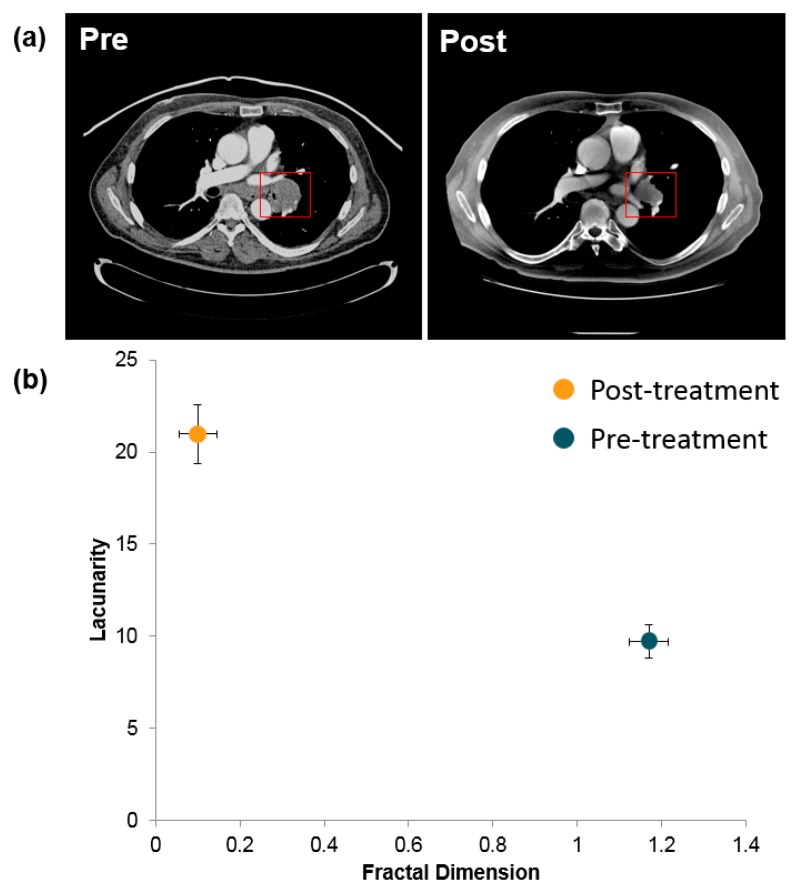
Lung CT images of a patient diagnosed with small cell lung carcinoma. (**a**) Pre- and post-CT images of the patent obtained before and after treatment. These images were loaded in Image J (Version 1.49), converted to 8 bit, and binarized; the background was set to white. The region of interest containing the tumor was defined manually. (**b**) Fractal dimension and lacunarity of primary small cell lung cancer (SCLC) tumors of three patients before and after treatment. The fractal dimension and lacunarity were calculated via the FracLac plugin (Box size was set from 0 pixels to 45% of image size, with 12 grid start positions using the grayscale differential scan). Significance was determined via t-test (*p* < 0.05).

FD and LC of three patients with SCLC pre- and post-treatment was used to evaluate their tumors on CT scans (Figure 1a). Before treatment, the scanned tumors had a high FD and a low LC, but this effect was reversed after treatment (*p* < 0.05) (Figure 1b).

### 3.2. IHC Staining and Fractal Analysis of Normal and Malignant SCLC Tissue.

**Figure 2:**
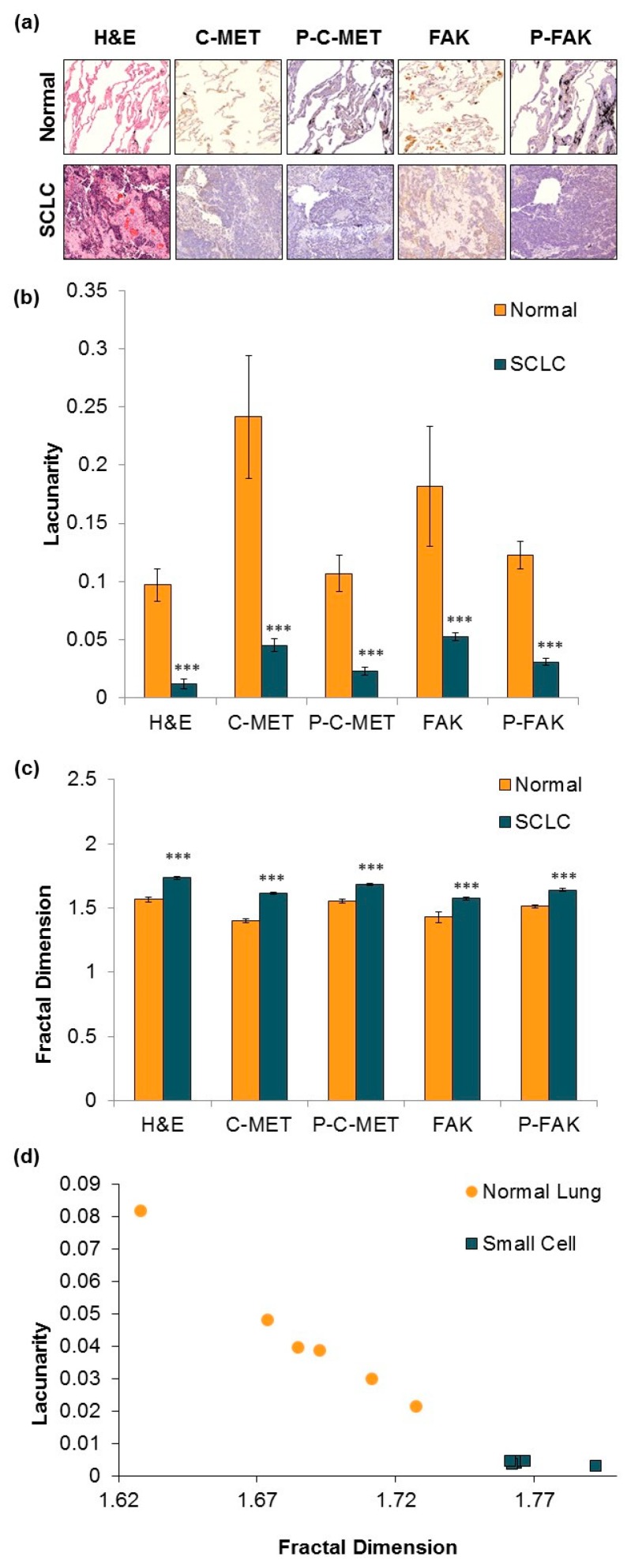
Immunohistochemistry images and fractal analysis of SCLC tissues. (**a**) Normal lung and SCLC tissue sections were processed for H&E and IHC staining with specific ab. (**b**) The fractal dimension of the stained normal (*n* = 6) and SCLC (*n* = 24) tissues was calculated using the FracLac plugin for ImageJ. Images were analyzed at 15×. Statistical significance was determined using an analysis of variance (ANOVA) with Tukey post-test. (∗ equals *p* ≤ 0.05, ∗∗ equals *p* ≤ 0.01, ∗∗∗ equals *p* ≤ 0.005). (**c**) The lacunarity of the stained normal (*n* = 6) and SCLC (*n* = 24) tissues was calculated using the FracLac plugin for ImageJ. Images were analyzed at 15×. Statistical significance was determined using an ANOVA with Tukey post-test. (∗ equals *p* ≤ 0.05, ∗∗ equals *p* ≤ 0.01, ∗∗∗ equals *p* ≤ 0.005). (**d**) Six areas of two formalin-fixed paraffin-embedded (FFPE) specimens (normal and SCLC) were individually analyzed and values of FD (*D*_B_) against lacunarity (*Λ*) were plotted for each region sampled. Images were analyzed at 40×.

A novel method that we had previously developed for differentiating tissue was utilized to identify the specific profiles of normal and SCLC tissue [13,14]. Tissues were stained with hematoxylin and eosin (H&E), c-MET, phospho c-MET, FAK, and phospho FAK staining, which were then scanned using a 3D-Histech Pannoramic SCAN whole slide scanner, and images were analyzed at 15× magnification using the ImageJ plugin FracLac to calculate the FD and LC (Figure 2a). The total number of tumors analyzed was 24 SCLC tissues and 6 normal lung tissues. SCLC tumor tissues had significantly higher FD and lower LC compared to normal lung tissue (Figure 2b,c; *p* < 0.01). This pattern was evident for all the antibodies used in this study, suggesting that FD or LC may offer a rapid and quantifiable method to distinguish between benign and malignant tissue for SCLC. As FD is not a unique identifier (i.e., different shapes can have similar FD), LC can be used to help differentiate these shapes (Figure 2d). The utilization of these two parameters in unison could serve as a future predictor of SCLC histology. 

### 3.3. Fractal Analysis of Mitochondrial Morphology in SCLC 

**Figure 3:**
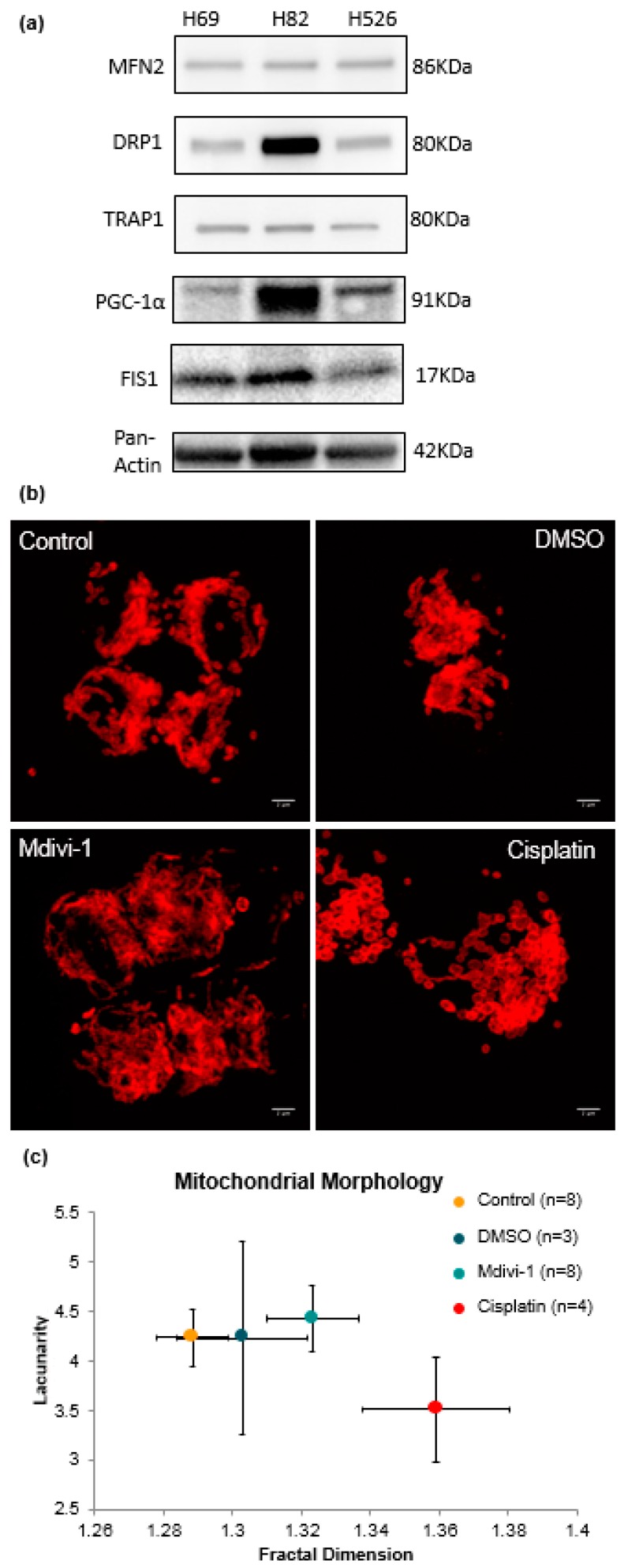
Mitochondrial protein expressions, immunofluorescence images, and fractal analysis of SCLC cell lines. (**a**) Representative immunoblots showing expression of mitochondrial proteins in a panel of SCLC cell lines. Protein loading was normalized by measurement of protein concentration. Three independent replicate experiments were performed. The full western blots are visualized in Supplementary Figure S1. (**b**) Mitochondrial morphology of H69 cells. Cells were treated for 4 h with 4 μM mdivi-1 (inhibitor of mitochondrial division dynamins), 4μM cisplatin, or equal volume of DMSO vehicle control. After fixation in 4% paraformaldehyde, cells were stained with TOM20. Two independent experiments were performed. In each case cells were selected at random for analysis. The number of cells in each experiment is indicated on panel (c). (**c**) Plot of individual values of FD (*D*_B_) against lacunarity (*Λ*) for each cell type.

The expression of mitochondrial dynamics proteins in SCLC cell lines was analyzed by immunoblotting (Figure 3a). The three SCLC cell lines H69, H82, and H526 were evaluated for the expression of mitochondrial fission and fusion proteins, including MFN2, DRP1, TRAP1α, PGC-1, and FIS1 [15,16]. DRP1 expression was higher in these cell lines compared to MFN2 fusion regulator. DRP1 was highly expressed in the H82 suspension SCLC cell line, more so than in the others. Protein-dependent mitochondrial morphology was evaluated by immunofluorescence staining with Tom20 antibody in untreated (control/DMSO) and treated (cisplatin) cells (Figure 3b). A total of eight control, three dimethylsulfoxide (DMSO)-treated, eight mdivi-1-treated and four cisplatin-treated H69 cells were visualized and evaluated for FD and LC using the FracLac plugin. Control cells had a number of fragmented and disjointed mitochondrial within the cytoplasm. The mitochondria densely packed the entire cytoplasm, as well as overlapping with the nucleus. Cells were treated for 4 h with 4 μM cisplatin, 4 μM mdivi-1, or equal volume of DMSO vehicle control. Visually, it could be inferred that mitochondrial fission in mdivi-1 treated cells was decreased, resulting in a more elongated network. These visual differences were then quantified using FD and LC measurements (Figure 3c). Fractal dimension was significantly different for those four conditions (F (3, 19) = 3.827, *p* = 0.0267). Cisplatin-treated cells showed increased FD as compared with control (*p* < 0.05). Mdivi-1 had an increase in the fractal dimension as compared with control, but did not show statistical significance. LC was not significantly different for those four conditions, due to the large variance. However, Figure 3c shows a trend that cisplatin-treated cells had less LC.

### 3.4. Metabolic Characteristics of SCLC Cells

**Figure 4:**
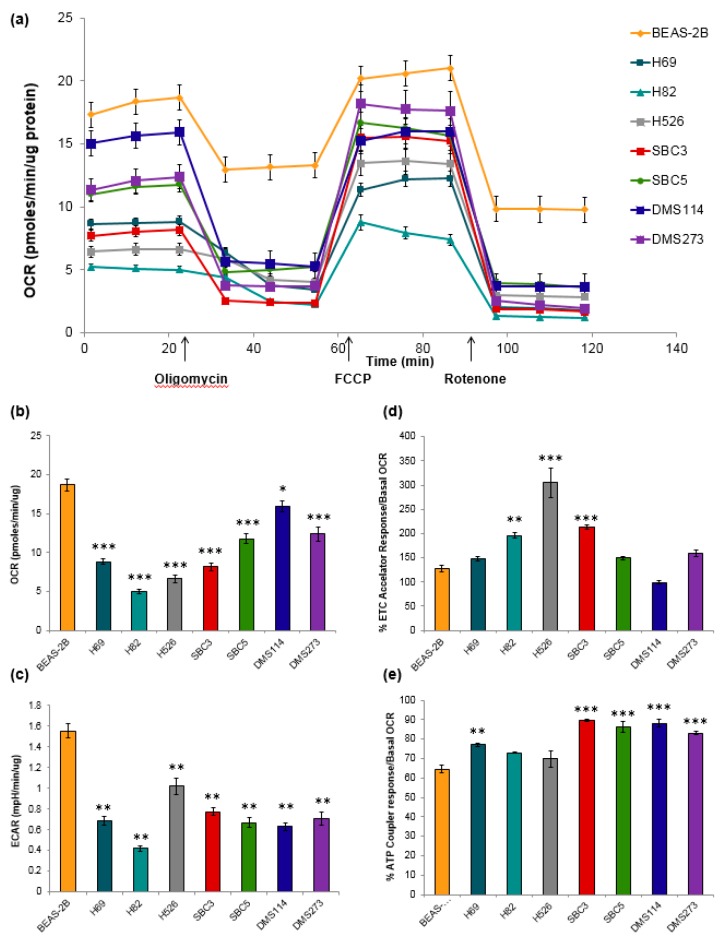
Mitochondrial oxidative and glycolytic activity in SCLC cell lines. Spare respiratory capacity indicates how close a cell operates to its bioenergetic limit. The mitochondrial stress test was used to obtain the bioenergetic parameters. (**a**) An example of one mitochondrial stress assay, arrows indicate the addition of 0.5 μM oligomycin A (oligo), 0.5 μM FCCP, and 1 μM rotenone. (**b**) Oxygen consumption rate (OCR), an indicator of mitochondrial oxidative phosphorylation. Basal mitochondrial OCRs in SCLC cells were significantly different compared to BEAS-2B OCR. (**c**) Extracellular acidification rate (ECAR), an indicator of glycolytic activity measured over time using Seahorse extracellular flux analyzer. Basal mitochondrial OCR in SCLC cells were significantly different compare to BEAS-2B. (**d**). Three SCLC cell lines (H82, H526, and SBC3) had significantly higher spare respiratory capacities compared to control BEAS-2B cells. Other SCLC cells lines had slightly increased capacities (H69, SBC5, DMS273) which were not significant, while DMS114 had a slightly decreased capacity. Results were analyzed via ANOVA with Tukey post-test (*p* < 0.001 ***, *p* < 0.01 **). Spare respiratory capacity was calculated using the mitochondrial stress test on the Seahorse Biosciences XF Analyzer as FCCP response/basal respiration. (**e**) Coupling efficiency of SCLC cell lines. SCLC cells lines demonstrated increased mitochondrial efficiency coupling respiration to ATP production. Results were analyzed via ANOVA with Tukey post-test (*p* < 0.001 ***, *p* < 0.01 **). Three independent replicate experiments were performed in quadruplicate for each experiment.

Mitochondrial activity has been described to be predicated upon the mitochondrial morphology during fission and fusion events within the cell. Fusion morphology has been described as elongated and networked to optimize energy output, meanwhile, fission morphology has fragmented features and a severed mitochondrial network that could be an indicator of mitochondrial dysfunction during removal of damaged components [17,18]. We examined the functional outputs of the various mitochondrial networks by measuring metabolic activity in SCLC cells. Oxidative phosphorylation and glycolysis were measured using oxygen consumption rate (OCR) and extracellular acidification rate (ECAR), respectively. An example of a typical mitochondrial stress test is shown in Figure 4a. Basal OCR was significantly lower in SCLC cell lines, compared to control BEAS-2B cells (Figure 4b). Basal ECAR, a measure of glycolytic activity, was significantly lower in the seven SCLC cell lines compared to BEAS-2B (Figure 4c). Metabolic phenotypes of SCLC cell lines were measured from the ratio of OCR to ECAR. A lower ratio indicates cells were more dependent on glycolysis compared to oxidative phosphorylation [19]. Reserve oxidative respiratory capacity was calculated as the difference between max OCR stimulated by FCCP treatment and basal OCR levels. Three SCLC cell lines (H82, H526, and SBC3) had significantly higher spare respiratory capacities compared to control BEAS-2B cells (Figure 4d). H69, SBC3, SBC5, DMS114, and DMS273 demonstrated increased mitochondrial efficiency coupling respiration to ATP production (Figure 4e). 

**Figure 5:**
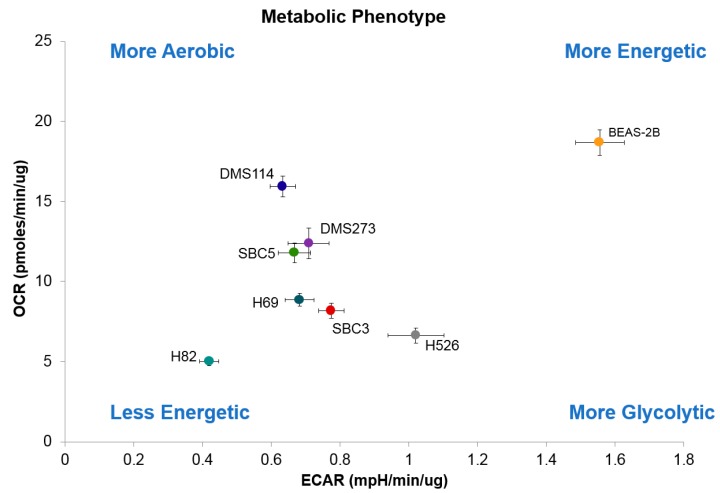
Bioenergetic profile of small cell lung cancer cell lines. Panel shows the ratios of OCR to ECAR in SCLC cells lines. A lower ratio indicates cells are more dependent on glycolysis compared to oxidative phosphorylation. Results are shown as the mean ± SEM of three independent experiments and were analyzed via ANOVA with Tukey’s post-test (* *p* < 0.05, ** *p* < 0.005, *** *p* < 0.0001).

Figure 5 places the cell lines on the OCR-ECAR plane and provides a snapshot of the basal bioenergetic profile of the cells and how they relate to the control BEAS-2B. H82 was the least energetic cell line (*p* < 0.05), while BEAS-2B utilized both oxidative phosphorylation (*p* < 0.05) and glycolysis (*p* < 0.0001) to meet their energetic needs. DMS114 was more aerobic (*p* < 0.05) while H526 was more glycolytic (*p* < 0.05).

### 3.5. Cytotoxicity of SCLC Cell Lines with Metformin and Mdivi-1

**Figure 6:**
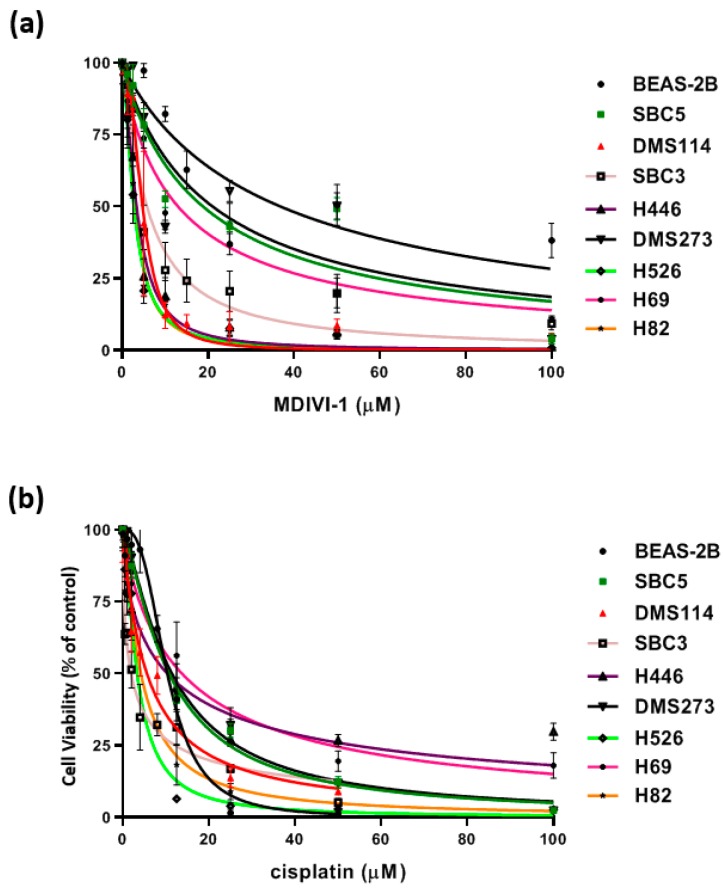
Cytotoxicity of SCLC cell lines to mdivi-1 and cisplatin. SCLC and control cells in 1% serum media were treated with increasing concentrations of **(a)** mdivi-1 and **(b)** cisplatin as indicated for 72 h, and cell viability was then assessed via calcein-AM uptake, as described. The results were normalized as a percentage of the DMSO treated controls and analyzed via a sigmoidal dose response curve using Prism software (version 5.0) to calculate the EC_50_ value for each drug. Three independent replicate experiments were performed for each experiment.

SCLC cell lines, H69, H82, H446, H526, SBC3, SBC5, DMS114, and DMS273, were evaluated for cytotoxicity with cisplatin, a standard chemotherapeutic agent for SCLC, and mitochondrial drug mdivi-1 (Figure 6). SCLC cell lines and control BEAS-2B cells in 1% serum media were treated with increasing concentrations of mdivi-1 and cisplatin, and cell viability was assessed via calcein-AM uptake. MYC was expressed in all cell lines but was not correlated with mdivi-1 cytotoxicity (Supplementary Table S1). The hydrolysis of calcein-AM by intracellular esterases produces calcein, a strongly fluorescent, hydrophilic compound that is well-retained in the cytoplasm. All eight SCLC cell lines were more sensitive to mdivi-1 than were the control BEAS-2B cells. In the case of cisplatin, four cell lines were more sensitive to this treatment, and four cell lines H69, H446, SBC5, and DMS273 had an EC_50_ value close to or higher than control BEAS-2B cells (Table 1). Cellular differences in response to cisplatin were comparable with clinical chemotherapy treatment, which for SCLC tumors often results in significant tumor shrinkage, however, relapse is usually quite rapid because of genetic and non-genetic tumor heterogeneity [20]. 

## 4. Discussion

SCLC has a high propensity for early metastases, and a deceptively positive initial response to cytotoxic chemotherapy that is often followed by acquired resistance [21]. Unlike in the case of non-small cell lung cancer, there are no tools for the early detection of SCLC [4,5,6]. Currently, CT imaging and biopsies are used to diagnose and monitor the disease, but these techniques offer a constrained view of early diagnosis, disease progression, and treatment monitoring. These efforts, and related investigations using serum biomarkers such as miRNA and CEA/chromogranin/NSE, have failed to produce validated biomarkers of early detection [22]. This complicates clinical management of SCLC, as the diagnosis occurs largely during late stages and the tumor morphology is ubiquitously pervasive with high metastatic potential [23,24]. In contrast to adenocarcinoma lung tumors, which grow more uniformly, compounded upon the primary tumor sites [25], SCLC is observed to exhibit invasive growth through the various alveoli, veins, and bronchi by expanding without preserving its central size or shape [26,27]. This leaves a layer of necrosis at the cancer origin site that cannot be easily quantified on 2D CT images. Furthermore, there is a dearth of genomic data in SCLC, primarily characterized by TP53 and RB1 alterations [28], which suggests that biomarkers should be pursued in areas of plentiful data such as CT scans, tissue immunohistochemical slides, and cell lines to determine how the self-similar and fractal nature of these images can act as a predictive model for clinical management. SCLC also has a unique fractal microenvironment that is self-similar at the cell level, tissue level, thoracic level, and metastatic site level, where the features of the metastatic tumors predicate themselves on the initial conditions of the primary site [27,29]. To demonstrate the clinical use of these measurements, we applied them across multiple scales from radiological to the cellular scale, where we measured the FD and LC of CT images, tissue, cells, and mitochondria. The data showed consistent correlations, providing strong evidence that macroscopic FD and LC measurements reflect differences in the tumor cells that give rise to them.

### 4.1. The Lung Is a Fractal Pattern

Fractals are mathematical constructs that show self-similarity over a range of scales and non-integer fractal dimensions [30]. Evaluation of FD and LC quantifies the irregular spatial patterns present in SCLC growth, which relates to specifics of the initial tumor and has the ability to predict its future course of evolution, as well as response to treatment [7,31]. This tool, when applied to image analysis, together with cellular and tissue analyses, could be useful in both early diagnosis and classification of the tumor in patients with SCLC. This study presented the fractal measurements of SCLC at different magnifications from cellular to tissue to cell lines, and the unique fractal properties were quantified utilizing FD and LC. The advantage of utilizing fractal geometry methods is that, unlike Euclidean methods, they are able to quantify and analyze complex, dimensionless structures through FD and LC measurements [9,30]. Fractal methodology has been used to quantify the complex lung physiology at various branching levels [32,33,34,35,36,37]. Since different tumors and lung characteristics, at a microscopic scale, will create different patterns of growth that are reflected at the macroscopic patterns of the tumor, FD and lacunarity (LC) can not only differentiate benign from malignant, but can also provide additional information on the biology of the tumor. 

The lung follows a branching fractal pattern where the various alveoli and bronchioles exhibit self-similarity across different scales [7,38,39]. Lung morphogenesis is an iterative process that begins with the bifurcation of the developing trachea into the left and right lung buds [39,40,41,42]. Following a sequential branching pattern, the lung buds grow and divide to form a fractal space-filling, tree-like architecture with 23 generations of branching [40,41,42]. Similarly, the pulmonary vasculature develops alongside the airways, and the gas exchange surfaces are formed on the peripheral generations of the branching system (Figure 1). The branching structure of the acinus has a slightly different branching structure to the conducting region of the lung, and its architecture is elegantly described by the Hilbert curve fractal [43]. However, images of the lung obtained at different magnifications exhibit self-similarity, thus, they are amenable to characterization and measurement using fractal geometry (Figure 1). From a functional perspective, the fractal branching pattern of the lung also regulates recruitment of the terminal airspaces from a previously unventilated compartment during inhalation [38,39]. Fractal branching is, therefore, a fundamental and crucial feature of lung development and function, and tumor development at a lower biological scale will be reflected in a disturbance in this fractal pattern.

### 4.2. Radiological Measurements of Fractal Geometry 

The alterations in lung structure that define the appearance of lung cancer in medical images are most often described in simple terms rather than the precise quantifiable measurements afforded by modern imaging and analysis tools [44,45]. For example, lung nodules are commonly characterized by size and volume, because other characteristics such as shape or CT density did not predict the presence of lung cancer in several studies, despite the intricately detailed information present in images [46,47,48,49]. However, several studies that evaluated the fractal texture analysis of solitary pulmonary nodules were able to statistically outperform radiologists for identification of lung nodules [50,51]. This discrepancy, we believe, is due in part to the use of classic Euclidean geometry, which distinguishes gross differences in geometry, such as volume, but there is information hidden in the complexity of the structure, such as texture and statistical properties of shape, that often goes uncaptured [50,51,52]. Thus, non-trivial measures of FD and LC of SCLC at the cellular level, tissue level, and organ level by quantifying cell lines, histological slides, and CT scans, are expected to exhibit a similar pattern. Pre-treatment CT scans were characterized by a high FD (high complexity) and low LC (homogeneous), while the post-treatment scans showed a low FD (low complexity) and high LC (more heterogeneity). We found these measurements to reflect the initial conditions of the tumor, its growth rate, and the condition of the lung. The initial features of the tumor, FD and LC, were affected in a similar pattern across a number of patients pre- and post-treatment. These self-similar morphological abnormalities are already under consideration of FD analysis to detect clinically relevant changes during lung cancer development and treatment in radiology [53,54,55,56].

### 4.3. Fractal Patterns of Tumor Tissue Differentiate SCLC

The fractal dimension box counting method has been capable of differentiating between malignant and normal tissue in a number of neoplastic studies involving hepatocellular carcinoma [57], endometrioid endometrial adenocarcinoma [58], oral squamous cell carcinoma [59], breast cancer [60,61], prostate adenocarcinoma [62], and renal cell carcinoma [63]. However, it was not until more recently that FD was utilized alongside LC as an additional parameter of fractal geometry to quantify colorectal adenocarcinomas [64,65], breast cancer [66], and cervical cancer [67]. Fractal analysis, while unable to differentiate individual shapes with a unique identifying number, can be utilized as a measure of homogeneity, with lower values indicating decreasing self-similarity in an image [57,68,69]. Fractal LC, meanwhile, analyzes the texture within the image beyond what can be observed with standard measurements, making it ideal to quantify the complexity of stained tissue [64,70,71]. Gheonea et al. demonstrated that FDs can be used to distinguish between malignant and benign histological images, and even to distinguish between primary and metastatic tumors within liver [71]. While fractal measurements have been met with success in other cancer types, this is the first study to apply these methods to SCLC. While other pathological markers, such as synaptophysin and chromogranin, are utilized to diagnose small cell lung cancer, more efforts are needed to differentiate the unique small cell lung cancer patterns that may correlate with treatment response and tumor burden. This makes FD and LC the ideal tools to differentiate normal tissue from malignant SCLC tissue as a potential diagnostic biomarker for SCLC. 

We observed that the FD values of all SCLC tissues stained for H&E, c-MET, phospho c-MET, FAK, and phospho-FAK were significantly higher than the FD values of normal tissue. One explanation may be that the SCLC tissue exhibits a more homogeneous fractal phenotype due to the self-similarity of the cancer clusters within the tissue. This is re-affirmed by the lower LC in SCLC tissue that indicates the low heterogeneity, while the high FD signifies their high complexity. However, these observations are unsurprising, as SCLC tissue is often characterized by densely packed, self-similar clusters with features of nuclear molding, finely granular (salt and pepper) chromatin, and scant delicate cytoplasm [72,73]. A higher FD has been shown to be associated with high distant metastatic risk in breast cancer tissue [66], but a high lacunarity was associated with lower metastatic risk, as observed in our normal lung tissue. These observations hint at a possibility that the fractal measurements of SCLC tissue may not only differentiate between normal and cancer tissue, but could potentially be shown to be a prognostic biomarker of progression and resistance. 

### 4.4. Dysfunction of Mitochondrial Morphology Correlates with Fission and Fusion Dynamics

At the sub-cellular level, mitochondria exist as a dynamic intracellular network, playing a vital role in cellular architecture and cellular metabolism that allows them to respond and adapt throughout the cell cycle and to withstand cell stresses such as increased energy demand, nutrient deprivation, or hypoxia [17,74]. Mitochondrial networks are often elongated, fragmented, or reticulated, making them ideal candidates to examine using fractal geometry. These classifications are indicative of the relative rates of fission and fusion occurring within the network, and may change depending on the state of the cell, especially in early stages of SCLC. The mitochondrial network is thought to be linked to the overall dysfunction of the mitochondria, which allows cancer cells to utilize irregular atypical energy production mechanisms. The mitochondrial network is also reliant on the balance between fission and fusion events that regulate mitochondrial dynamics, or the movement of mitochondria along the cytoskeleton and alteration of their morphology. Cancer cell metabolism and mitochondrial dynamics have been gaining traction as a viable emerging therapeutic target, and mitochondrial DNA has been shown to be a requirement for carcinogenesis [75,76,77].

In our study, gain of the reserve respiratory capacity correlated to cellular resistance to stress, and the coupling efficiency indicates the proportion of respiratory activity involved in ATP production. Our results show a correlation in SCLC cell line between increased glycolysis rate and attenuated respiration capacity, as classically first observed by Warburg and termed the Warburg Effect [78,79]. This metabolic switch allows cancer cells to produce the energy required for proliferation. However, the unique feature of SCLC is that it is not only highly proliferative, but has an adaptive metabolic plasticity that allows it to preserve its metastatic potential as it travels throughout the organism [80]. This aspect is compounded in SCLC cells that go through a reiterative process of forming dense clusters of tumor cells that penetrate the blood barrier and enter the blood stream as a cluster rather than individual cells [81,82], similar to observations in another highly aggressive cancer: inflammatory breast cancer [83]. We observed that LC of the mitochondria decreased with treatment, indicating lowered heterogeneity of the mitochondrial network, and the increased fractal dimension could hint at a possible increase in complexity due to mitochondrial damage. Therefore, the metabolic uptake of the cell lines and the observed morphological changes in the mitochondria predicate the tumor’s future ability for mitogenesis and motogenesis, as was also observed on the CT scan images.

## Figures and Tables

**Table 1:** EC_50_ value for various SCLC cell lines.

Drug EC_50_	Beas-2B (Control)	H69	H82	H446	H526	SBC3	SBC5	DMS114	DMS273
mdivi-1 μM	36.9	13.01	2.889	3.356	2.738	5.763	18.83	4.735	21.07
Cisplatin μM	10.37	13.76	4.218	10.37	3.357	1.940	10.76	5.626	11.44

## Supplementary Materials

The following are available online at https://www.mdpi.com/2077-0383/8/7/1038/s1, Table S1: MYC status in SCLC cell lines.
