# Supplementary material for: Small Cell Lung Cancer Therapeutic Responses Through Fractal Measurements: From Radiology to Mitochondrial Biology
Source: J Clin Med. 2019 Jul 16;8(7):1038. doi: 10.3390/jcm8071038 (PMC6679065; doi:10.3390/jcm8071038)
Supplement: Supplementary file 1 [file jcm-08-01038-s001.zip › jcm-520424-supplementary.pptx]

## Slide 1
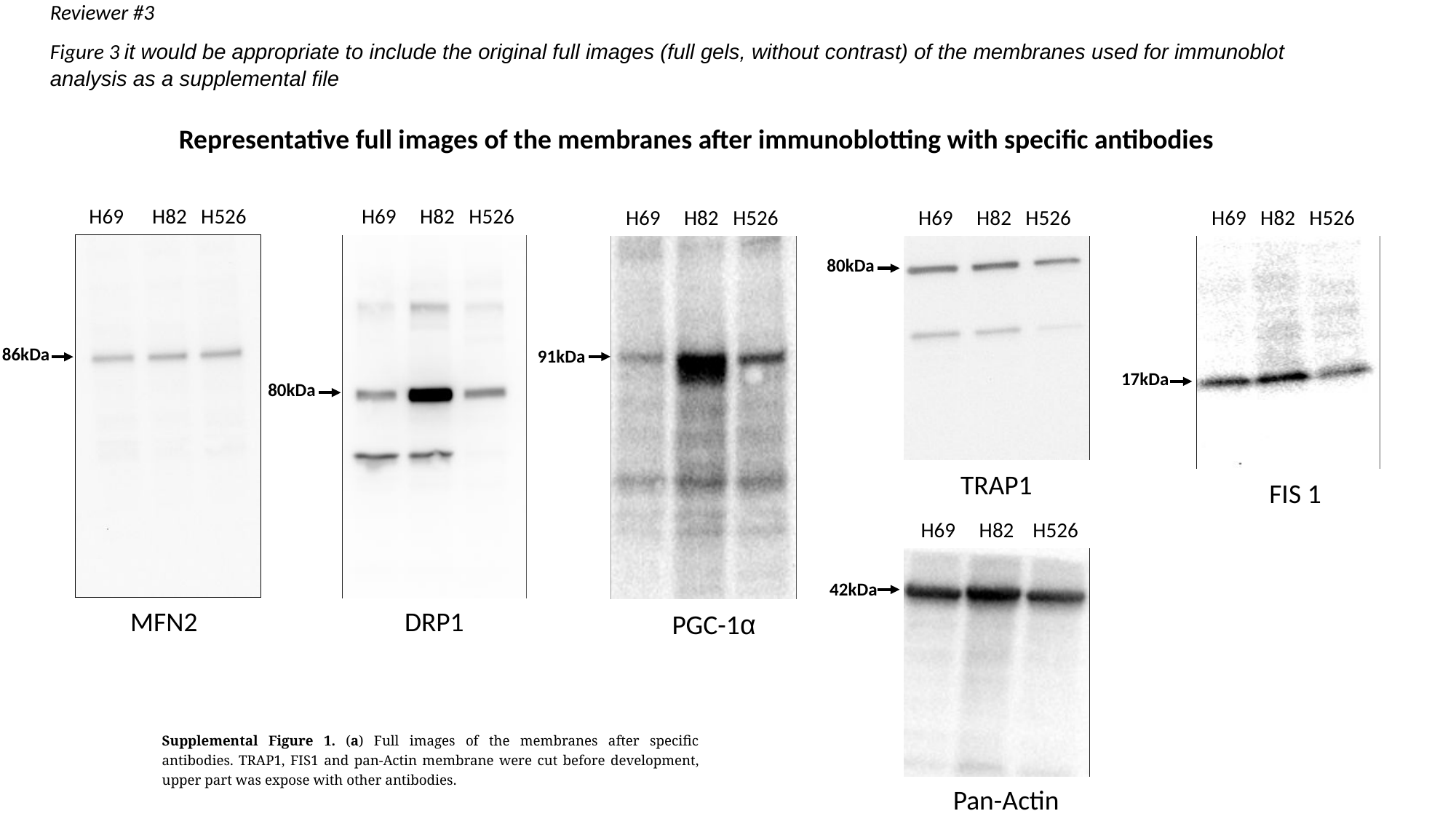

Reviewer #3
Figure 3 it would be appropriate to include the original full images (full gels, without contrast) of the membranes used for immunoblot analysis as a supplemental file
Representative full images of the membranes after immunoblotting with specific antibodies
H69 H82 H526
86kDa
MFN2
 H69 H82 H526
80kDa
DRP1
 H69 H82 H526
91kDa
PGC-1α
 H69 H82 H526
80kDa
TRAP1
 H69 H82 H526
17kDa
FIS 1
H69 H82 H526
42kDa
Pan-Actin
Supplemental Figure 1. (a) Full images of the membranes after specific antibodies. TRAP1, FIS1 and pan-Actin membrane were cut before development, upper part was expose with other antibodies.
