# Supplementary material for: Small Cell Lung Cancer Therapeutic Responses Through Fractal Measurements: From Radiology to Mitochondrial Biology
Source: J Clin Med. 2019 Jul 16;8(7):1038. doi: 10.3390/jcm8071038 (PMC6679065; doi:10.3390/jcm8071038)
Supplement: Supplementary file 1 [file jcm-08-01038-s001.zip › jcm-520424-supplementary.docx]

**Supplemental Material**

**Supplemental Table 1.** MYC status in SCLC cell lines

| **Cell line** | **Mdivi-1 IC50 (µM)** | **MYC Status (amplification)** | **References** |
| --- | --- | --- | --- |
| H69 | 13.01 | N-myc | https://doi.org/10.18632/oncotarget.8826 |
| H82 | 2.889 | C-myc | www.atcc.org |
| H446 | 3.356 | C-myc | www.atcc.org |
| H526 | 2.738 | N-myc | https://doi.org/10.18632/oncotarget.8826 |
| SBC3 | 5.763 | C-myc | DOI: Published May 1989. Takahashi et al. |
| SBC5 | 18.83 | C-myc | doi: 10.1186/s12885-015-1202-4 |
| DMS114 | 4.735 | C-myc | https://doi.org/10.1158/1078-0432.CCR-15-2448 |
| DMS273 | 21.07 | C-myc | https://doi.org/10.1093/jnci/djw122 |
